# Biotin Enhances Testosterone Production in Mice and Their Testis-Derived Cells

**DOI:** 10.3390/nu14224761

**Published:** 2022-11-10

**Authors:** Kota Shiozawa, Misato Maeda, Hsin-Jung Ho, Tomoko Katsurai, Md. Zakir Hossain Howlader, Kimiko Horiuchi, Yumi Sugita, Yusuke Ohsaki, Afifah Zahra Agista, Tomoko Goto, Michio Komai, Hitoshi Shirakawa

**Affiliations:** 1 Laboratory of Nutrition, Graduate School of Agricultural Science, Tohoku University, 468-1, Aramaki Aza Aoba, Aoba-ku, Sendai 980-8572, Japan; 2 Department of Biochemistry and Molecular Biology, University of Dhaka, Dhaka 1000, Bangladesh; 3 International Education and Research Center for Food and Agricultural Immunology, Graduate School of Agricultural Science, Tohoku University, 468-1, Aramaki Aza Aoba, Aoba-ku, Sendai 980-8572, Japan

**Keywords:** biotin, testis, testosterone, adenylate cyclase

## Abstract

Late-onset hypogonadism, a male age-related syndrome characterized by a decline in testosterone production in the testes, is commonly treated with testosterone replacement therapy, which has adverse side effects. Therefore, an alternative treatment is highly sought. Supplementation of a high dosage of biotin, a water-soluble vitamin that functions as a coenzyme for carboxylases involved in carbohydrate, lipid, and amino acid metabolism, has been shown to influence testis functions. However, the involvement of biotin in testis steroidogenesis has not been well clarified. In this study, we examined the effect of biotin on testosterone levels in mice and testis-derived cells. In mice, intraperitoneal treatment with biotin (1.5 mg/kg body weight) enhanced testosterone levels in the serum and testes, without elevating serum levels of pituitary luteinizing hormone. To investigate the mechanism in which biotin increased the testosterone level, mice testis-derived I-10 cells were used. The cells treated with biotin increased testosterone production in a dose- and time-dependent manner. Biotin treatment elevated intracellular cyclic adenosine monophosphate levels via adenylate cyclase activation, followed by the activation of protein kinase A and testosterone production. These results suggest that biotin may have the potential to improve age-related male syndromes associated with declining testosterone production.

## 1. Introduction

Late-onset hypogonadism (LOH) is a male age-related syndrome characterized by a decline in testosterone production. Blood levels of testosterone peak in 20- to 30-year-old males and then decline with age [1]. Decreased testosterone not only reduces the frequency of sexual behavior, but also induces lifestyle-related diseases such as obesity, diabetes, sarcopenia, and osteoporosis [2,3]. It also has a significant impact on mental health and can increase the incidence of depression [4].

Testosterone, in biological males, is produced by the Leydig cells of the testes and has both anabolic and androgenic effects. Leydig cells are the major testosterone-producing cell type, in which pituitary luteinizing hormone (LH) binds to the LH receptor and activates adenylate cyclase (ADCY). ADCY converts ATP into 3′, 5′-cyclic adenosine monophosphate (cAMP), which activates protein kinase A (PKA), and cAMP response element-binding protein (CREB) and other steroidogenic enzymes [5,6,7]. CREB is a crucial transcription factor for the regulation of the genes involved in steroidogenesis such as steroidogenic acute regulatory protein (StAR) and cytochrome P450 cholesterol side chain cleavage enzyme (CYP11A). Cholesterol is transferred into the mitochondria by StAR and converted to pregnenolone by CYP11A in the inner mitochondrial membrane. Pregnenolone is converted to progesterone and testosterone in the smooth endoplasmic reticulum [5].

Suppressive approaches targeting declining testosterone production in the testes could help to extend healthy life expectancy. Male hormone replacement has been used to alleviate LOH symptoms but is reported to be associated with several side effects, warranting the need to develop alternative therapies. Vitamins and other dietary ingredients have attracted attention as safe candidates, and some have been reported to increase testosterone production [6,7,8,9,10,11,12,13]. Biotin is a water-soluble vitamin that functions as a cofactor for carboxylases related to the metabolism of carbohydrates, lipids, and amino acids, from microorganisms to higher organisms [14]. In animals, biotin deficiency causes dermatitis and alopecia, as well as impairment of bone formation, fetal development [15], and female gonad morphology [16,17]. These observations have been illustrated by experiments using egg white, a source of dietary protein containing avidin, and germ-free animals to exclude biotin supply derived from intestinal microorganisms.

Additionally, studies have been conducted in which high doses of biotin were administered to experimental animals. Pharmacological doses of biotin have been shown to ameliorate diabetes and hypertension [18,19,20,21,22]. Several studies showed that extremely high doses of biotin influence hepatic and testis morphology, sperm quality, and spermatogenesis [23,24,25,26]. However, the involvement of administered biotin on steroidogenesis in testes has not been clarified well. In the present study, we investigated the effect of biotin on testosterone production in mice and their testis-derived tumor cells in which steroidogenesis is regulated by the cAMP-PKA pathway [5]. We found that biotin treatment enhances testosterone production in mice and in testis-derived cells, and in the case of cells via elevation of cAMP levels.

## 2. Materials and Methods

### 2.1. Materials

Biotin was obtained from the FUJIFILM Wako Pure Chemical Corporation (Osaka, Japan). Biotin for cell culture experiments was dissolved in ethanol at 4 mM for the stock solution and stored at −20 °C until use. Forskolin (Fsk, an activator of adenylate cyclase), N-[2-(p-bromo cinnamyl amine) ethyl]-5-isoquinoline sulfonamide dihydrochloride (H-89, an inhibitor of PKA), MDL-12,330A (MDL, an inhibitor of adenylate cyclase; Sigma-Aldrich, St. Louis, MO, USA), and 3-isobutyl-1-methyl-xanthine (IBMX, an inhibitor of phosphodiesterase; Cayman Chemical, Ann Arbor, MI, USA) were dissolved in dimethyl sulfoxide (Sigma-Aldrich) to prepare a stock solution of 10 mM. 

### 2.2. Animal Experiments

Male BALB/c mice (CLEA Japan Inc., Tokyo, Japan), 7 weeks old, were maintained at a room temperature of 23 ± 2 °C, relative humidity of 50 ± 10%, and a 12 h light/dark cycle, with free access to a F-2 laboratory diet (Funabashi Farm Co., Funabashi, Japan) and water. Mice in the biotin group received intraperitoneal injections of biotin (1.5 mg/kg B.W.), the same dosage as in a previous study [22], and those in the control group received saline (n = 7/group). According to our previous study [13], the mice were euthanized 6 h after biotin administration, and blood and testes were collected. This study and all other procedures were approved and guided by the Animal Ethics Committee of Tohoku University. 

### 2.3. Cell Culture

Mice testis-derived Leydig cell culture I-10 cells (RIKEN BRC, Tsukuba, Japan) and MA-10 cells (ATCC, Manassas, VA, USA) were maintained in a nutrient mixture F-10 Ham (Sigma-Aldrich) supplemented with 10% fetal bovine serum (Biowest, Nuaillé, France), 50 U/mL penicillin, and 50  µg/mL streptomycin (Gibco, Thermo Fisher Science, Tokyo, Japan). Cells were maintained at 37 °C and 5% CO_2_/95% air, as controlled by the incubator. The maximum levels of biotin and additive agents were determined at concentrations that did not affect cell viability, using the Premix WST-1 Cell Proliferation Assay System (Takara Bio, Shiga, Japan).

### 2.4. Measurement of Testosterone, Progesterone, and Luteinizing Hormone Levels

For cell cultures, testosterone and progesterone levels were sampled from the medium after culturing with or without biotin. I-10 cells or MA-10 cells were cultured at 6.0 × 10^4^ cells/well in 12-well plates and incubated for 24 h. The culture medium was replaced with a new medium containing biotin (final concentrations of 0–40 µM). After 0–24 h, the culture medium was collected and centrifuged at 1000× *g* for 5 min. Testosterone and progesterone levels in the supernatant of the medium were determined using enzyme-linked immunosorbent assay (ELISA) kits (testosterone, #582701; progesterone, #582601; Cayman Chemical). Because both cells produce a relatively higher level of progesterone compared with testosterone, for testosterone measurements, the collected media were used directly, and for progesterone measurements, the collected media were diluted 20 times by ELISA buffer attached to the ELISA kit. 

The blood and testes were processed for measurement by ELISA. Blood was centrifuged at 1870× *g* for 15 min to obtain the serum from the supernatant. The testes (100 mg) were homogenized in fifty volumes of phosphate-buffered saline (PBS). Hormones from the serum (10 μL) and homogenized testes (500 μL) were extracted with 10 volumes of diethyl ether and centrifuged at 1870× *g* for 3 min. At least 80% of the diethyl ether layer was collected, and the process was repeated twice. The ether layer fraction was then centrifuged and evaporated using a vacuum evaporator (Spin Dryer Light VC-36R; TAITEC Corp., Saitama, Japan). The extract was resuspended (serum, 200 μL; testes, 100 μL) and diluted with the ELISA buffer (serum, twice; testes, 100 times). Serum luteinizing hormone (LH) levels were determined using LH ELISA kits (#ERKR7010, Endocrine Technologies, Newark, CA, USA). 

### 2.5. Measurement of cAMP Levels

I-10 cells were cultured at 6.0 × 10^4^ cells/well in 12-well plates and incubated for 24 h. The culture medium was replaced with a fresh medium containing biotin (final concentrations of 0 or 40 µM). After 1 h, the cells were washed twice with PBS, then 0.1 M HCl was added for cell lysis. cAMP levels in the cell lysates were determined using a cAMP ELISA kit (#581001, Cayman Chemical). 

### 2.6. Reporter Gene and RNA Interference Assays

The CREB-mediated reporter plasmid (pCREB-Luc, Clontech Laboratories, Inc., Mountain View, CA, USA) and the transfection control plasmid, pmiwZ (encoding *E. coli* β-galactosidase [27], from the Japanese Cancer Research Resources Bank, Ibaraki, Japan), were transfected into I-10 cells using the FuGENE HD Transfection Reagent (Promega, Madison, WI, USA) in OPTI-MEM-1 medium (Gibco, Thermo Fisher Science) according to the previously described procedure [7]. A fresh medium (F-10 Ham medium) was added 24 h after transfection, and the cells were incubated for another 24 h. The cells were then treated with Fsk or biotin for 3 h. After preparing the cell lysate using Passive Lysis Buffer (Promega), the activities of luciferase and β-galactosidase in cell lysates were measured according to the method described in our previous experiments [7]. Reporter gene activity was normalized to the β-galactosidase activity.

Double-stranded siRNAs, targeting adenylate cyclase 9 (*Adcy9*), sodium-dependent multivitamin transporter (*Slc5a6*), and Negative Control Medium GC Duplex #2 were purchased from Invitrogen (Thermo Fisher Scientific). The siRNA sequences are shown in Table 1. I-10 cells were transfected with siRNAs using Lipofectamine RNAiMAX Reagent (Invitrogen, Thermo Fisher Scientific) for 24 h, according to the reagent manufacturer’s instructions and our previous experiment [28].

### 2.7. mRNA Quantification

To extract total RNA from cells, we used an Isogen acid phenol-guanidine thiocyanate-based reagent (Nippon Gene, Tokyo, Japan). cDNA was synthesized from 4 µg of isolated RNA using previously described procedures [28]. PCR was performed using an ABI 7300 Real-Time PCR system (Applied Biosystems, Foster City, CA, USA) and SYBR Premix EX Taq (Takara Bio). The primer sequences used for PCR are listed in Table 2. mRNA expression levels were normalized to those of eukaryotic translation elongation factor 1α1 (*Eef1*α*1*).

### 2.8. Measurement of Biotin Levels

I-10 cells were cultured at 6.0 × 10^4^ cells/well in 12-well plates and incubated for 24 h. The culture medium was replaced with a new medium containing biotin (final concentration of 0–40 µM). After 24 h, cell lysates were collected. Free biotin levels in cell lysates were measured using *Lactobacillus plantarum* (ATCC8014) following the method described in [29]. Briefly, the bacterial solution and medium for the biotin assay (Nissui Pharmaceutical Co., Ltd., Tokyo, Japan) were dispensed in a 96-well plate. Cell lysate sample solutions or standard biotin solutions at specific concentrations were added to the wells. After 24 h of incubation at 37 °C, the absorbance of culture media was measured using a spectrophotometer and biotin levels were calculated using a standard curve.

### 2.9. Statistical Analysis

Data are presented as mean ± standard error. Study statistics include Student’s *t*-test, one-way ANOVA followed by Dunnett’s test, and two-way repeated-measures ANOVA followed by Tukey–Kramer’s multiple comparisons test. Statistical analyses were performed using StatcelQC software (OMS Publishing, Saitama, Japan). Statistical significance was indicated in each figure.

## 3. Results

### 3.1. Effect of Biotin on Testes and Serum Testosterone Levels in Mice

**Figure 1:**
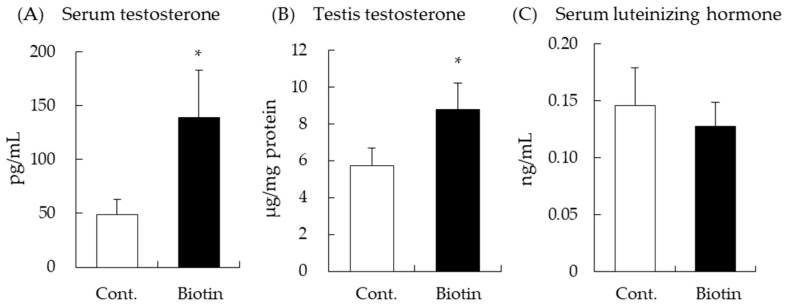
Intraperitoneally administered biotin increases testosterone production in BALB/c mice. Testosterone levels in serum (**A**) and testis (**B**), and serum luteinizing hormone levels (**C**) were measured by ELISA. Data are presented as mean ± standard error (n = 4–7). * *p* ≤ 0.05, vs. control (Cont.) group, Student’s *t*-test.

Serum and testicular testosterone levels were determined by ELISA after intraperitoneal injection of biotin (1.5 mg/kg of body weight) for 6 h. Serum testosterone levels increased with biotin treatment (*p* = 0.05, vs. control group, Figure 1A). In addition, testosterone levels in the testes were significantly higher in the biotin group than in the control group (Figure 1B). LH secreted from pituitary gland increases testosterone production in testes, but there was no significant difference in LH levels between the biotin and control groups (Figure 1C). These results suggest that biotin enhances testosterone production in the testes and serum without elevating serum LH.

### 3.2. Biotin Stimulates Testosterone Production in Testis-Derived cells

**Figure 2:**
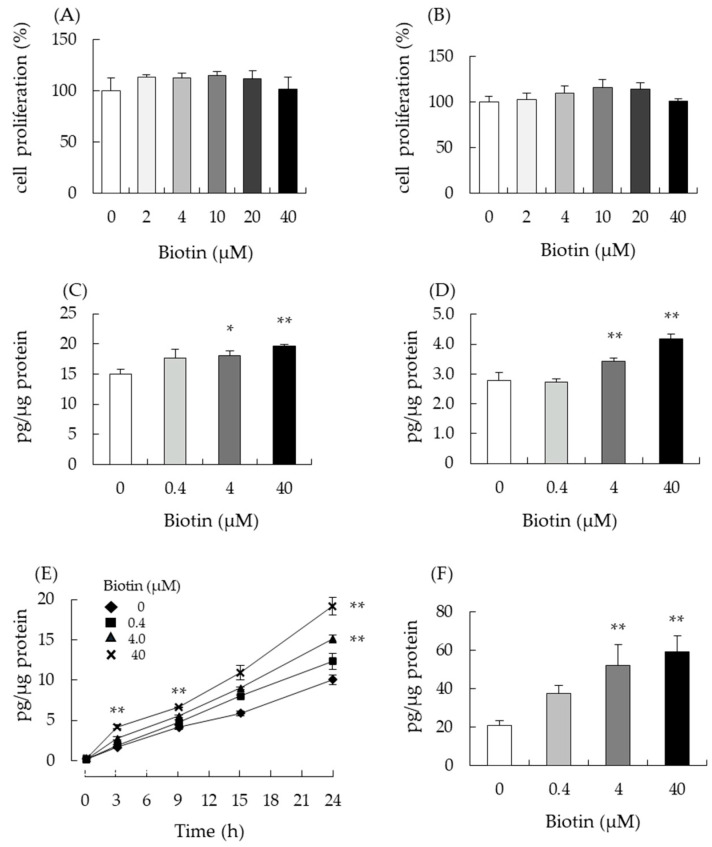
Biotin enhances testosterone production in mouse testis-derived tumor cells. The effect of 24 h of biotin treatment on cell proliferation of I-10 (**A**) and MA-10 cells (**B**) were evaluated by WST-1 assay. Testosterone levels in cultured media, as measured by ELISA after 24 h treatment with biotin, are shown for I-10 cells (**C**) and MA-10 (**D**). The same was done for progesterone levels in the media after 24 h biotin treatment of I-10 cells (**F**). (**E**) Testosterone levels of I-10 cells in a cultured medium after biotin treatment (0, 3, 9, 15, and 24 h) were measured by ELISA. Data are presented as mean ± standard error (n = 3). * *p* < 0.05, ** *p* < 0.01 vs. 0 µM biotin group, Tukey–Kramer test.

Next, we analyzed the effect of biotin on testosterone production in mouse testis-derived I-10 and MA-10 cells. Both cells synthesize and secrete testosterone depending on the cAMP-PKA pathway. First, we used the WST-1 assay to determine the maximum biotin concentration that did not cause cytotoxicity in either cell line. Neither I-10 nor MA-10 cells showed significant changes in cell viability or proliferation at biotin concentrations up to 40 µM (Figure 2A,B). Next, we measured testosterone levels in the culture medium to determine whether biotin modulated testosterone production. After 24 h of biotin treatment, testosterone levels in the media of I-10 and MA-10 cells increased in a dose-dependent manner (0–40 µM, Figure 2C,D). In the time-course analysis, biotin significantly enhanced testosterone levels in the medium at 3 and 9 h of treatment (40 µM) in I-10 cells (Figure 2E). Progesterone, a precursor of testosterone, is also synthesized and secreted from I-10 cells. Progesterone levels in the culture medium were significantly elevated by biotin treatment for 24 h at 4 µM or 40 µM in I-10 cells (Figure 2F). These results indicated that biotin could enhance steroidogenesis in both I-10 and MA-10 testis-derived cells.

### 3.3. Biotin Stimulates the cAMP/PKA Pathway for Testosterone Synthesis

**Figure 3:**
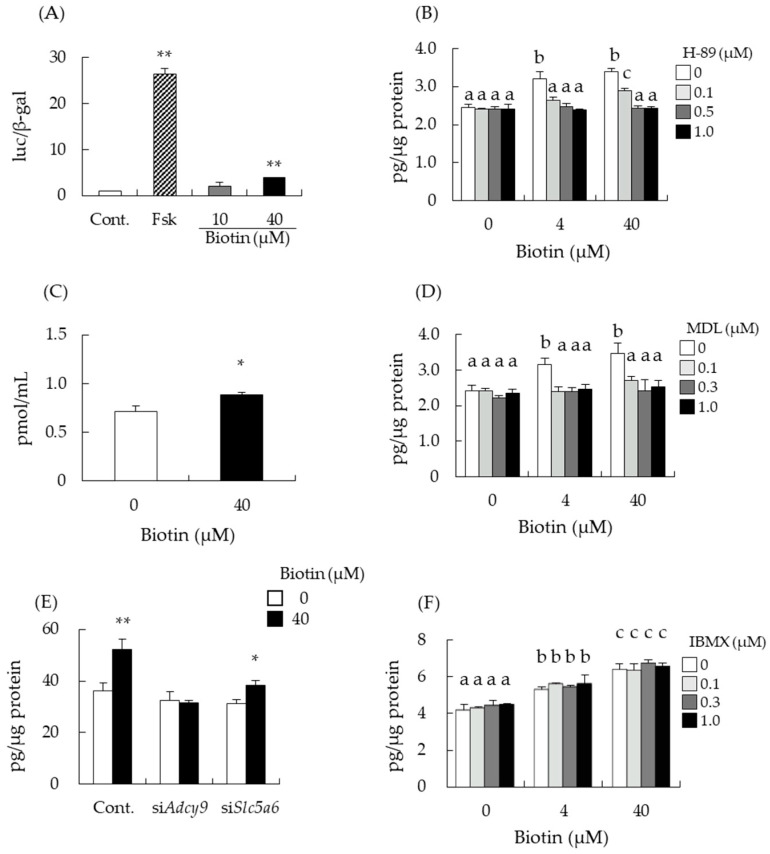
Biotin stimulates testosterone production via the cAMP/PKA pathway. CREB-mediated reporter activities were measured 3 h after the treatment of forskolin (Fsk), an activator of ADCY, or biotin (**A**). The testosterone levels in the I-10 cells cultured medium with/without biotin and PKA inhibitor H-89 for 24 h were measured by ELISA (**B**). cAMP levels in I-10 cell lysate after the treatment of biotin for 1 h were measured by ELISA (**C**). The testosterone levels in the I-10 cell culture medium with/without biotin, ADCY inhibitor MDL (**D**) and PDE inhibitor IBMX (**F**), were measured by ELISA after 24 h of treatment. The testosterone levels of the medium from I-10 cells, transfected with siRNAs targeting *Adcy9* or *Slc5a6*, were also measured (**E**). Data are presented as mean ± standard error (n = 3). Values with different letters are significantly different at *p* < 0.05 vs. the 0 µM biotin group, Tukey–Kramer. * *p* < 0.05, ** *p* < 0.01 vs. the control (Cont.) or the 0 µM biotin group, Dunnett’s test or Student’s *t*-test.

Steroidogenesis in I-10 and MA-10 cells is regulated by the cAMP-PKA pathway [1]. Therefore, we evaluated whether biotin stimulated PKA activation in a reporter gene assay mediated by CREB, which is a crucial transcription factor involved in steroidogenesis activated by PKA. Luciferase activity of CREB reporter gene was significantly increased by biotin treatment in I-10 cells, indicating the upregulation of CREB activity (Figure 3A). Next, we investigated the effects of cAMP/PKA pathway inhibitors on biotin-mediated enhancement of testosterone synthesis in I-10 cells. H-89 is a well-known PKA inhibitor. In the absence of biotin, treatment with H-89 at 0 to 1 µM did not affect testosterone levels in the medium of I-10 cells (Figure 3B). However, in the presence of biotin (4 µM or 40 µM), H-89 abolished the enhancement of testosterone levels by biotin. As PKA is a protein kinase activated by increasing intracellular cAMP levels, cAMP levels were measured with ELISA. cAMP levels in the cell lysate were significantly increased by biotin treatment for 1 h at 40 µM (Figure 3C). Intracellular cAMP levels are regulated by adenylate cyclase (ADCY) and cyclic nucleotide phosphodiesterase (PDE). Simultaneous treatment with MDL-12,330A (MDL), an ADCY inhibitor, suppressed biotin-mediated enhancement of testosterone levels (Figure 3D). Mice have 10 types of *Adcy* genes, of which, *Adcy9* mRNA was dominantly expressed in I-10 cells (Supplemental Figure S1). Thus, we employed siRNA transfection in I-10 cells to downregulate *Adcy9* expression. Treatment with siRNA-targeted *Adcy9* abolished the biotin-mediated enhancement of testosterone levels (Figure 3E). In contrast, treatment with IBMX, a non-specific inhibitor of PDE, did not modulate the enhancement of testosterone levels by biotin treatment (Figure 3F). These results suggest that biotin stimulates testosterone production by increasing cAMP levels through ADCY activation.

### 3.4. Intracellular Transport of Biotin Did Not Contribute to the Enhancement of Testosterone Levels

**Figure 4:**
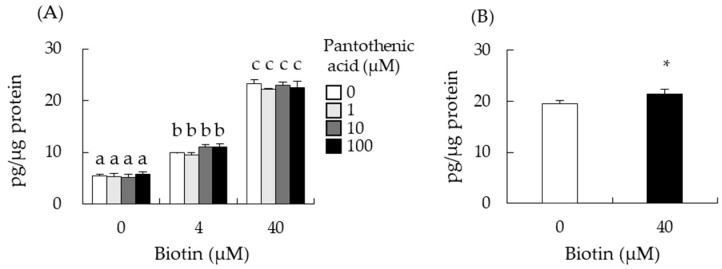
Intracellular transport of biotin did not contribute to the enhancement of testosterone levels. The testosterone levels in the I-10 cells cultured medium with/without biotin and pantothenic acid for 24 h were measured by ELISA (**A**). Free biotin levels in I-10 cell lysates after 24 h of biotin treatment were measured by bioassay using *Lactobacillus plantarum* (ATCC8014) (**B**). Data are presented as mean ± standard error (n = 3). Values with different letters are significantly different at *p* < 0.05 vs. 0 µM biotin group, Tukey–Kramer. * *p* < 0.05, vs. 0 µM biotin group, Student’s *t*-test.

Biotin is transported into cells through a membrane transporter called the sodium-dependent multivitamin transporter (SMVT, SLC5A6) [14]. Although I-10 cells were treated with siRNA targeting *Slc5a6*, biotin still increased testosterone levels (Figure 3E). SLC5A6 can transport not only biotin but also other nutrients such as pantothenic acid [30], and pantothenic acid may interfere with biotin transport to the cells. Simultaneous treatment of biotin with pantothenic acid (0–100 µM) did not affect testosterone enhancement by biotin in I-10 cells (Figure 4A). We also measured free biotin levels in I-10 cells 24 h after biotin treatment. Free biotin levels in I-10 cells were significantly increased (9.7%) by biotin treatment compared with those of the control (Figure 4B). These data suggest that the uptake and elevation of intracellular biotin levels through SLC5A6 do not contribute to the enhancement of testosterone levels in I-10 cells.

### 3.5. Effect of Biotin-Analogues on Testosterone Levels in I-10 Cells

**Figure 5:**
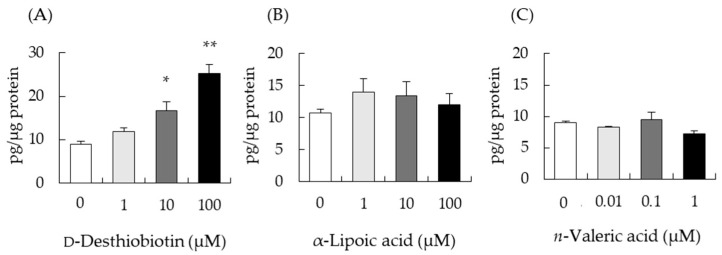
The effect of biotin-analogues on testosterone production in I-10 cells, 24 h post-treatment. Shown are the testosterone levels in I-10 cell cultured media, treated with d-desthiobiotin (**A**), α-lipoic acid (**B**), or *n*-valeric acid (**C**) for 24 h, measured by ELISA. Data are presented as mean ± standard error (n = 3). * *p* < 0.01, ** *p* < 0.0 vs. 0 µM group, Tukey–Kramer.

The structure of biotin consists of the rings of thiophene and imidazole, and valeric acid as a side chain. To compare which structural features impact the enhancement of testosterone levels, we analyzed the stimulatory capacity of three biotin-related compounds, d-desthiobiotin, α-lipoic acid, and *n*-valeric acid. Testosterone levels were unchanged from treatment with α-lipoic acid or *n*-valeric acid, but they were significantly increased by d-desthiobiotin in a dose-dependent manner (Figure 5). These results suggest that the imidazole ring of biotin, rather than the thiophene ring, may be important for enhancing testosterone production.

## 4. Discussion

In this study, we found that intraperitoneal biotin administration increased the testosterone levels in the serum and testes of male BALB/c mice (Figure 1A,B). In contrast to our findings, Pastén-Hidalgo et al. recently reported that the addition of a pharmacological dose of biotin diet (97.5 mg/kg diet) for 8 weeks induced the increase of spermatogonia layers and a loss of seminiferous tubule lumen in the testes of male BALB/cAnNHsd mice but did not alter serum testosterone levels [24]. This raises the possibility that the steroidogenic effect of biotin is a dose-dependent occurrence.

Leydig cells in the testis are steroidogenic, and testosterone synthesis is tightly regulated by LH secreted from the anterior pituitary gland, which is a part of the hypothalamic-pituitary-testicular axis [5]. We measured serum LH levels after intraperitoneal injection of biotin, which increased testosterone production without changing LH levels (Figure 1C). This suggests that biotin increases testosterone production by directly acting on the steroidogenic cells in the testis. Several studies have indicated that food ingredients including vitamins can regulate testosterone production in a similar manner. Dietary supplementation of menaquinone-4, a type of vitamin K2, and intraperitoneal administration of S-allyl cysteine could stimulate testosterone production in male rats and mice, respectively, without the alternation of blood LH levels [6,13]. On the other hand, ginger, onion, and their extracts have been demonstrated to show enhancement of testosterone production in animal experiments with or without the modulation of LH levels [31,32].

Various other food ingredients and pathways have been identified to promote testosterone production in steroidogenic cells [11]. Horigome et al. showed that 5,7-dimethoxyflavone (DMF) extracted from black ginger *Kaempferia parviflora* and nobiletin, which is found in citrus fruits and has a similar structure with DMF, enhances testosterone production via the inhibition of PDE [10]. Sulfur-containing amino acids, cysteine sulfoxides, and S-allyl cysteine promoted the phosphorylation of PKA and its downstream CREB. This resulted in enhanced progesterone and testosterone production in I-10 cells. These reports pointed out that the elevation of steroid production is due to not only the increase of intracellular cAMP but also cAMP-independent activation pathways of PKA and CREB [12,13]. We hypothesized that biotin’s testosterone-increasing effect was achieved via the activation of a similar pathway.

Mouse testis-derived I-10 was used to examine the involvement of the cAMP-PKA pathway and CREB activation in the testosterone-enhancing effect of biotin treatment in this study. I-10 and MA-10 cells are known to have properties similar to Leydig cells and can synthesize testosterone depending on cAMP levels in cells [5]. Biotin is shown to increase testosterone production in these cell lines as well (Figure 2). Salazar-Anzures et al. reported that the biotin-supplemented diet augmented protein levels of the c-KIT and active forms of ERK and AKT in mice testes [25]. The signaling pathway that includes c-KIT, ERK, and AKT enhances cell proliferation, and the activation of these signaling molecules may lead to morphological changes in the testes. Since the active form of ERK can stimulate the activation of CREB and modulate the transcription of CREB target genes [33,34], the activation of ERK by biotin may stimulate testosterone production via CREB activation. This hypothesis is in line with our findings, in which the treatment of I-10 cells with PKA and ADCY inhibitors and siRNA-targeting *Adcy9* abolished biotin-induced enhancement of testosterone (Figure 3). Other food compounds, such as menaquinone-4, and its side-chain structural moiety, geranylgeraniol (GGOH), have also been observed to stimulate testosterone production by the activation of PKA [6,7]. In the case of GGOH, the enhancement of steroid production was abolished by *Adcy9* knockdown in I-10 cells, similar to the current study on biotin.

Biotin was reported to increase testosterone levels despite the treatment of I-10 cells with siRNA targeting *Slc5a6* (Figure 3E). SLC5A6 can transport not only biotin but also pantothenic acid [30], and pantothenic acid may interfere with biotin transport to the cells. However, Figure 4 indicated that extracellular biotin, not incorporated biotin, might be important to enhance steroidogenesis in this cell for activation of ADCY. Elevation of intracellular cAMP levels is the most crucial event for the promotion of steroidogenesis in Leydig cells. In this study, we show for the first time that biotin increases intracellular cAMP levels by activating ADCY. Several studies have reported that biotin can modulate intracellular ATP and secondary messenger levels, thereby regulating cell function [19,35,36,37]. Sone et al. indicated that biotin increases insulin secretion from the pancreatic islets of Langerhans by increasing ATP levels [19]. Biotin has also been shown to increase cGMP levels by activating guanylate cyclase, resulting in the elevation of ATP content, followed by the enhancement of insulin secretion and glucokinase mRNA expression in rat pancreatic islets [36], and the phosphorylation (activation) of AMPK and the phosphorylation (inactivation) of acetyl-CoA carboxylase, thus decreasing fatty acid synthesis in mice adipose tissue [37]. Biotinyl-AMP derived from biotin is thought to be the molecule that activates guanylate cyclase to increase cGMP levels [38]. d-Desthiobiotin, an intermediate of biotin biosynthesis in microorganisms, also enhanced testosterone production (Figure 5), suggesting that the activation of ADCY by biotin uses a different mechanism from that of guanylate cyclase.

**Figure 6:**
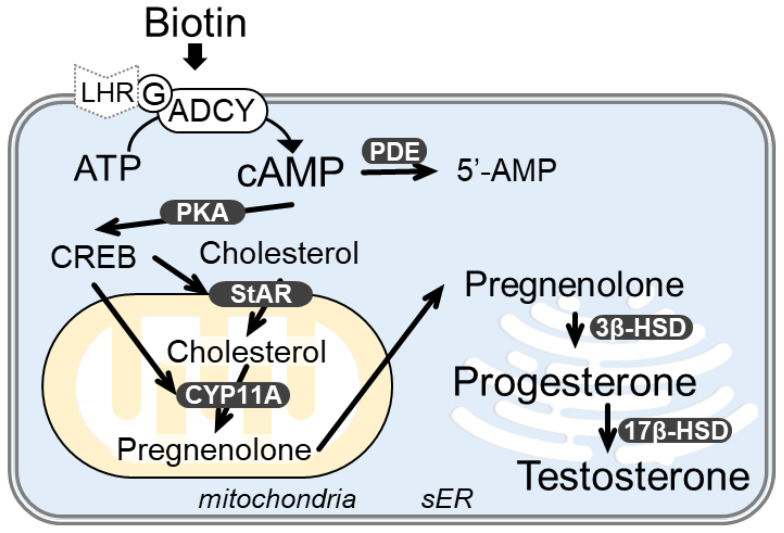
Presumed mechanism of biotin on the enhancement of testosterone production in I-10 cells. 3β-HSD, 3β-hydroxysteroid dehydrogenase; 17β-HSD, 17β-hydroxysteroid dehydrogenase; ADCY, adenylate cyclase; cAMP, 3′, 5′-cyclic adenosine monophosphate; CREB, cAMP response element-binding protein; CYP11A, cytochrome P450 cholesterol side chain cleavage enzyme; PKA, protein kinase A; sER, smooth endoplasmic reticulum; StAR, steroidogenic acute regulatory protein.

There are two types of pathways for ADCY activation: direct or via G protein-coupled receptors. The common structure of biotin and d-desthiobiotin may directly activate ADCY. Another possibility is that an unknown G protein-coupled receptor recognizes and binds biotin, which may activate ADCY. These results suggest that biotin activates ADCY and, consequently, the cAMP-PKA pathway to increase testosterone levels (Figure 6). However, the mechanism underlying ADCY activation and steroidogenic pathway modulation, including the events in the mitochondria and smooth endoplasmic reticulum by biotin, needs to be elucidated in the future. The clarifications on the effects of biotin on the expressions and activities of StAR, 3β-hydroxysteroid dehydrogenase, and 17β-hydroxysteroid dehydrogenase are especially important due to their rate-limiting ability on testosterone production [5,39,40,41]. These studies may clarify novel molecular mechanisms of biotin treatment and lead to further biotin-based therapeutic strategies in age-related diseases including LOH.

## Figures and Tables

**Table 1:** Double-stranded siRNA sequence.

Gene Name	siRNA Sequence	Accession No.
*Adcy9*	5′-CAUAGGAGUAGAAGAGGCCAGUGAA-3′	NM_009624.3
*Slc5a6*	5′-GAGUACCUAGAGCUCCGCUUCAAUA-3′	NM_001177621.1

**Table 2:** Nucleotide sequences of primers used for qRT-PCR.

Gene Name	Forward Primer Reverse Primer	Product Size (bp)	Accession No.
*Adcy1*	5′-GGTCCAGTGTTTTCCAGGGT-3′	100	NM_009622.22
	5′-CACCACACAGCCTTGAGCTA-3′		
*Adcy2*	5′-TCAACCCCAAGGGAGAAAGAC-3′	64	NM_153534.2
	5′-CCATCCAGAGTGTGTCGAGG-3′		
*Adcy3*	5′-GGAAAAGGACTCTCCTATGGTGG-3′	80	NM_138305.3
	5′-GCCTGCTGTCAGTGCCATT-3′		
*Adcy4*	5′-ATTGCTGCGTGTTGGGTTTC-3′	89	NM_001361604.1
	5′-CACCAGCCACAGCAGAAGTA-3′		
*Adcy6*	5′-TTCCTTTGGAAGCAGCTCGG-3′	56	NM_001368413.2
	5′-ATGGCATTGGTGCAGAGGAA-3′		
*Adcy7*	5′-CAGGGTATTAAGGTCCCAGCC-3′	91	NM_001037723.3
	5′-GACATCTTCTTCCCTGGCTCT-3′		
*Adcy8*	5′-TCATGATCGCCATCTACGCC-3′	99	NM_009623.2
	5′-TCCCCAGGAAATCTTCTCCAC-3′		
*Adcy9*	5′-CCTGTGTCAGGACAGTTCCATT-3′	51	NM_009624.3
	5′-TTCTGTGCTGAGTCCAAGGG-3′		
*Adcy10*	5′-AGAGCTCGACTCGTACCTGG-3′	86	NM_173029.3
	5′-CTCTGTGGTGGTCGAGGTTT-3′		
*Slc5a6*	5′-AGTGAATCAGGCTCAGGTGC-3′	71	NM_001177621.1
	5′-CATAGCAGGAGAGCACAGCA-3′		
*Eef1* *α1*	5′-GATGGCCCCAAATTCTTGAAG-3′	52	NM_010106.2
	5′-GGACCATGTCAACAATGGCAG-3′		

## Data Availability

Not applicable.

## Supplementary Materials

The following supporting information can be downloaded at https://www.mdpi.com/article/10.3390/nu14224761/s1, Supplementary Figure S1: mRNA expression levels of *Adcys* in I-10 cells as measured by qRT-PCR.
