# Supplementary figures and images for: Biotin Enhances Testosterone Production in Mice and Their Testis-Derived Cells
Source: Nutrients. 2022 Nov 10;14(22):4761. doi: 10.3390/nu14224761 (PMC9697070; doi:10.3390/nu14224761)

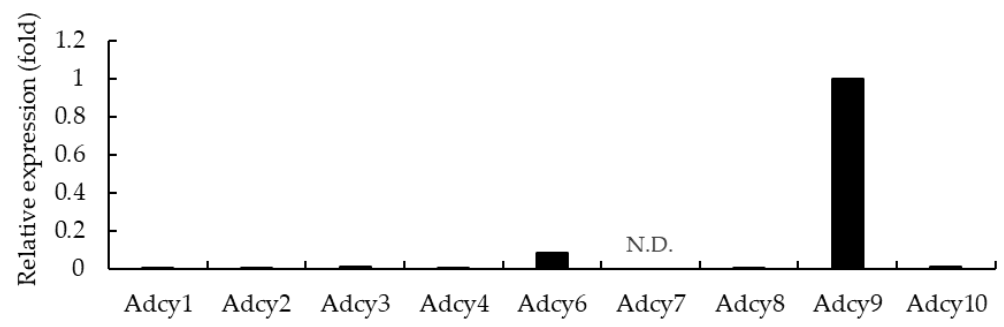

**Figure S1.** mRNA expression levels of *Adcys* in I-10 cells as measured by qRT-PCR.

Supplement: Supplementary file 1 [file nutrients-14-04761-s001.zip › nutrients-1949887-supplementary.pdf]
